# Dynamics in Quaternary Ionic Liquids with Non-Flexible Anions: Insights from Mechanical Spectroscopy

**DOI:** 10.3390/ijms241311046

**Published:** 2023-07-03

**Authors:** Oriele Palumbo, Annalisa Paolone, Frederik Philippi, Daniel Rauber, Tom Welton

**Affiliations:** 1 Istituto dei Sistemi Complessi, Consiglio Nazionale delle Ricerche, Piazzale A. Moro 5, I-00185 Rome, Italy; annalisa.paolone@roma1.infn.it; 2 Department of Chemistry, Molecular Sciences Research Hub, Imperial College London, White City Campus, London W12 0BZ, UK; f.philippi18@imperial.ac.uk (F.P.); t.welton@imperial.ac.uk (T.W.); 3 Department of Chemistry, Saarland University, Campus B 2.2, 66123 Saarbrücken, Germany; daniel.rauber@uni-saarland.de

**Keywords:** mechanical spectroscopy, ionic liquids, flexibility, relaxation

## Abstract

The present work investigates how mechanical properties and ion dynamics in ionic liquids (ILs) can be affected by ILs’ design while considering possible relationships between different mechanical and transport properties. Specifically, we study mechanical properties of quaternary ionic liquids with rigid anions by means of Dynamical Mechanical Analysis (DMA). We are able to relate the DMA results to the rheological and transport properties provided by viscosity, conductivity, and diffusion coefficient measurements. A good agreement is found in the temperature dependence of different variables described by the Vogel−Fulcher−Tammann model. In particular, the mechanical spectra of all the measured liquids showed the occurrence of a relaxation, for which the analysis suggested its attribution to a diffusive process, which becomes evident when the ion dynamics are not affected by the fast structural reorganization of flexible anions on a local level.

## 1. Introduction

Ionic liquids (ILs) have attracted huge interest due to their properties [1,2], which enable a wide range of possible applications [3,4], including catalysis and electrochemistry [5,6]. Moreover, these properties are tunable by means of a proper choice of the constituent ions. The initial progress in the design of ionic liquids was based on empirical structure–property relationships, but a new strategy is based on targeted modification [7]. The latter is a general methodology to enable purposeful design using well-defined changes to just one isolated variable [7,8,9] and focusing on properties of general importance [7,8]. This method could be a powerful tool to reduce the number of empirical studies to a small number of carefully selected promising candidates [7,8].

In this framework, it has been recently reported that [8,9] fluorination alone is not enough to obtain low viscosity, but a major role is played by conformational flexibility [9]. In particular, the conformational flexibility of anions is a key parameter to obtain lower values for viscosities and thermal transition temperatures and, at the same time, faster transport properties. Moreover, anion conformational flexibility is involved in the dynamics occurring within the ILs, and it has also been hypothesized that anion conformational relaxation correlates with translational diffusion [9,10,11,12].

Indeed, the dynamic behavior of ILs, typically classified as fragile glass formers [13], has been widely studied by several techniques [13,14,15,16,17,18,19], and the temperature dependence of the relaxation time in the liquid phase is reported to be well-approximated by the empirical Vogel−Fulcher−Tammann (VFT) equation [14,15,20,21]. In particular, a hopping process due to a translational, oscillatory motion of anions and cations relative to each other as they exchange partners in ion pairs has been reported by dielectric relaxation, a method that was also able to detect the occurrence of a correlated rearrangement of the molecular network [22,23]. Moreover, the measurement of diffusion coefficients corroborated the idea that any relative motion of two oppositely charged ions within the bulk liquid is not a simple sliding movement but involves more complex intramolecular rearrangements [10,13,22,23]. In imidazolium-based ILs, dielectric measurements also showed the presence of a secondary Johari and Goldstein relaxation attributed to a common butyl group [24]. Similar relaxations have also been observed below the glass transition temperature in phosphonium-based ILs [25]. More recently, several authors have focused on the occurrence of possible relationships between the timescales associated with ion diffusion and structural relaxations, which are probed by different techniques [18,19,26,27]. In this framework, a deep understanding of how the ion dynamics behave at different length scales is fundamental, and the dependence on ion compositions and, therefore, on the intermolecular interactions, should not be neglected. In particular, it has been reported both theoretically [19,27] and experimentally [18] that possible mesoscale aggregates influence the local and mesoscale dynamics and transport properties, which can be more or less correlated on different length scales, leading to different results depending on the physical property of interest.

The influence aggregates have on ion dynamics has also been reported by recent Dynamical Mechanical Analysis (DMA) measurements on several ILs composed of quaternary cations and two anions with different conformational flexibility [12]. These measurements showed the presence of a relaxation process in the liquid phase of the ILs with the flexible [NTf_2_]^−^ anion, which was related to the ion hopping between non-equivalent configurations; on the contrary, for other ILs with rigid ions, a fast dynamic at the local level in the liquid phase was rarely observed because a partial transition to a solid state is favored. Indeed, tetraalkylphosphonium cations give rise to ionic interactions between the oppositely charged ions, which can induce strong structural correlations, thus promoting the occurrence of nanoscale structural heterogeneities [12,28]. The presence of these aggregates, for which the organization can result in domain formation, hinders the observation of any relaxation in the liquid state and assists the occurrence of at least partial solidification [12,29].

Despite the large number of studies carried out, there is still demand for fundamental insight into ion dynamics to enable IL design and to inform how observations from different techniques can be rationalized with a general model. In this framework, the present study reports a DMA study of quaternary ionic liquids with non-flexible anions by comparing DMA results with the rheological and transport properties provided by viscosity, conductivity, and diffusion coefficient measurements.

**Figure 1:**
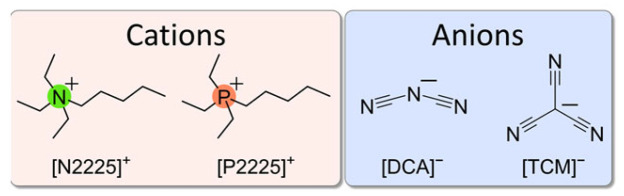
Molecular structures and abbreviations of the cations and anions used for the ILs.

ILs with quaternary cations are attracting considerable interest [30,31,32,33,34], but they are still less studied than the more common imidazolium- or pyrrolidinium-based ILs. In particular, phosphonium-based ILs have lower viscosity but higher thermal stability and electrical conductivity compared to their ammonium analogues, and these properties make them attractive for energy storage devices that require electrochemical stability at higher voltages (>3 V) [12,32,35]. The physical differences between phosphonium and ammonium cations, which differ only in the central N or P atom, have been attributed to the flexibility of the phosphonium cations [36] and to a more facile rotation around the P–C bond [8,36]. In particular, the cations considered in the present paper are triethylpentyl ammonium [N2225]^+^ and triethylpentyl phosphonium [P2225]^+^ (see Figure 1 for their molecular structures). They were coupled with two non-flexible anions, tricyanomethanide [TCM] and dicyanamide [DCA] (Figure 1).

The choice of rigid anions, which have fewer degrees of freedom for relaxation [8,33], is aimed at avoiding the fast structural reorganization of flexible anions on a local level, which could affect the ion dynamics. Indeed, previous mechanical spectroscopy investigations in combination with the calculation of ab initio PES [9] have pointed to the possible rate-limiting steps underlying structural relaxation, because conformational flexibility can provide alternative pathways for relaxation on an intermediate timescale. Moreover, in a series of ILs with the same imidazolium cations and various anions with different flexibility, DMA experiments [12] were able to highlight the role of the conformational flexibility in the relaxation detected in the liquid phase, as well as the differences observable in this relaxation when a rigid ion, such as [B(CN)_4_]^−^, is involved [12].

Indeed, the obtained results indicate that when the ion dynamics are not affected by the fast structural reorganization of flexible anions on a local level the relaxation dynamics measured by DMA show a diffusive character. The data analysis suggests that the occurrence of translational motion by means of hopping processes is possibly coupled to the rotational motions and to the transport properties.

## 2. Results and Discussion

### 2.1. Thermal Transitions and Density

**Figure 2:**
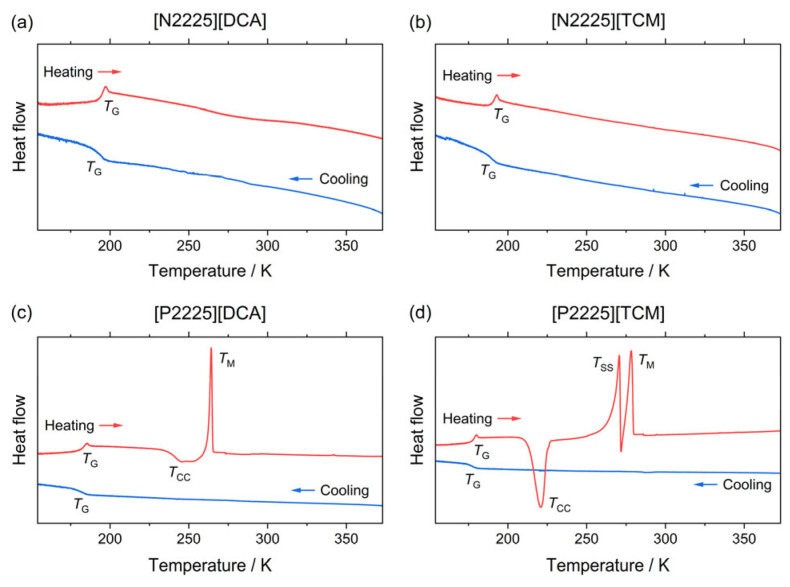
DSC traces of the ammonium (**a**,**b**) and phosphonium ionic liquids (**c**,**d**) with indicated thermal transitions (G: glass transition; CC: coldcrystallization upon heating; SS: solid–solid transition; M: melting). Blue curves relate to the initial cooling, and red ones relate to the subsequent heating step. All the curves are given in exo down representation.

The thermal behavior of the four liquids is characterized by DSC measurements (Figure 2). All the studied liquids (see curves in Figure 2) display upon cooling the typical feature of a glass transition, and the relative transition temperatures [T_G_] are reported in Table 1.

The glass transition temperatures displayed by the phosphonium-based ILs are lower than those of their ammonium analogues, showing little difference (in the order of 6%). An even smaller difference is observed between liquids with the same cation but a different anion, because the two considered anions are both rigid; as such, no contribution coming from the different flexibilities is observable, in contrast to different series of ammonium of phosphonium ILs previously reported [33,34], which listed anions with different flexibilities. Upon heating, the ammonium-based ILs revert from the glassy to the liquid state (Figure 2), while the two phosphonium samples undergo additional cold crystallization and subsequent melting upon further heating.

The temperatures observed for the cold crystallization and the melting of the studied samples are reported in Table 1 for comparison. It is worth noting that the [P2225][TCM] sample displays an exothermic peak just before melting, which is likely due to the occurrence of a solid–solid phase transition, as already observed in other similar systems [33]. Indeed, it is widely accepted that ILs can display different polymorphs in their crystal state, and that these phases can also depend upon the crystallization conditions [33,37].

The temperature-dependent density values were also measured for all four ILs; the data and the parameters obtained from the best linear fit are reported in the Supplementary Materials, Tables S1 and S2. The values obtained at room temperature are reported in Table 1 as a reference. These data indicate that the liquids with the [DCA]^−^ anion have higher density than their [TCM]^−^ analogues, while the liquids with the phosphonium cation have a higher density than those with the ammonium analogues. This behavior is in agreement with similar results obtained for phosphonium and ammonium ILs with a rigid anion, such as [B(CN)_4_]^−^ [33], while it is the opposite to what is observed in systems where the quaternary cations are combined with flexible fluorinated anions. Indeed, in the latter case, the flexible phosphonium ionic liquids showed a decrease in density compared to the more rigid ammonium analogues as a result of the larger free volume [33].

### 2.2. Viscosity

**Figure 3:**
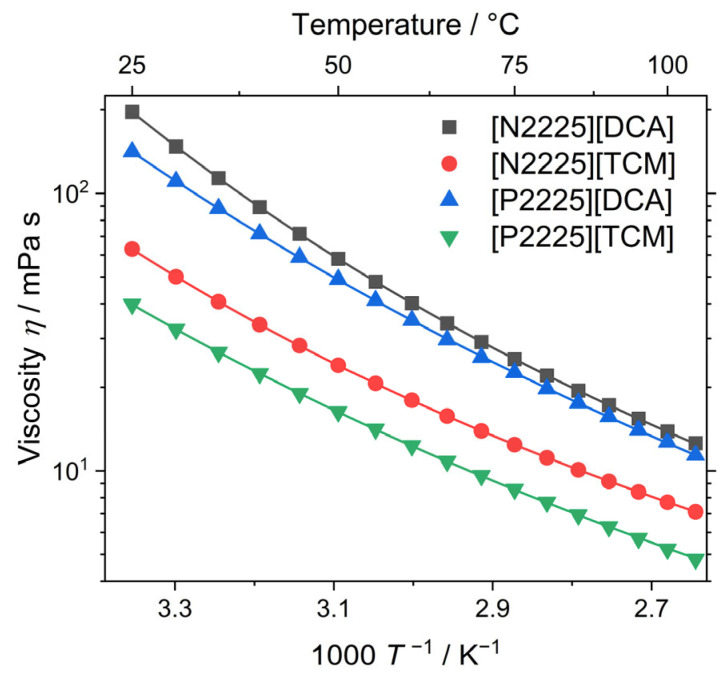
Experimental viscosity values of the investigated ionic liquids (symbols). Drawn lines are the fittings according to the VFT equation (Equation (1)).

The viscosity η of ionic liquids is a central limitation for mass and heat transport. For the vast majority of practical applications, low viscosities are desirable to improve performance. For the presently studied ILs, the viscosities as well as the Vogel–Fulcher–Tammann (VFT) fitting parameters (Equation (1)) and Angell’s strength factor for temperature-dependent viscosity are given in Table 2 and plotted in Figure 3. The experimental values and the activation energies for the viscous flow according to the Arrhenius equation (Equation (2)) at 25 °C are given in the Supplementary Materials (Table S3).

At 25 °C, the viscosities for the ammonium ionic liquids are higher than those of the phosphonium analogues for a given cation, while the [DCA]^–^ samples have significantly higher viscosities than the [TCM]^–^ ionic liquids. The trend of the higher viscosity of ammonium ionic liquids compared to the isostructural phosphonium is a quite general finding, and this has also been observed for the bis(trifluoromethanesulfonyl)imide anion [NTf_2_]^–^ (also termed [TFSI]^–^), with both comparably small [38,39] and comparably large cations [40], as well as for the tetracyanoborate [B(CN)_4_]^−^ anion [33]. This is rationalized by the stronger interactions in the ammonium samples [35] resulting from the lower flexibility of the ions [41]. Lower viscosities of [TCM]^−^-based ionic liquids compared to [DCA]^−^-based analogues have also been observed for the 1-buty-1-methyl-pyrrolidinium cation [42]. For samples with the 1-ethyl-3-methyl imidazolium cation, only minor differences in viscosity for the two anions have been found [43].

The temperature-dependent viscosity reveals that the order of magnitude of the viscosity remains the same in the experimental temperature range. Due to the temperature dependence of the activation energy for the viscous flow, fitting with the phenomenological VFT equation needs to be applied, as shown in Figure 3. The temperature dependence of transport properties Y for glass-forming materials, such as ionic liquids, can be quantified by Angell’s strength parameter $\delta_{Y}$ ($\delta_{Y}=B_{Y}/T_{0,\eta }$). Therefore, low values of $\delta_{Y}$ indicate a highly temperature-dependent activation energy, which is found for so-called ‘fragile’ liquids, while ‘strong’ liquids, on the contrary, have a constant activation energy and high values of $\delta_{Y}$ [44]. For the investigated ammonium and phosphonium ionic liquids, the fragility of the ammonium samples with the same anion is higher than for the phosphonium samples, and the samples with the [TCM]^−^ anion are more fragile than the [DCA]^−^-based ones. All of these ionic liquids with cyano-based anions are highly fragile. For comparison, the $\delta_{Y}$ of [P2225][NTf2] has been given as 6.32 [45], while the commonly used cations 1-butyl-3-methylimidazolium and 1-buty-1-methylpyrrolidinium with the [DCA]^−^ anion have been reported to have $\delta_{Y}$ values of 7.24 and 3.24 [41,45].

### 2.3. Conductivity and Walden Plot

**Figure 4:**
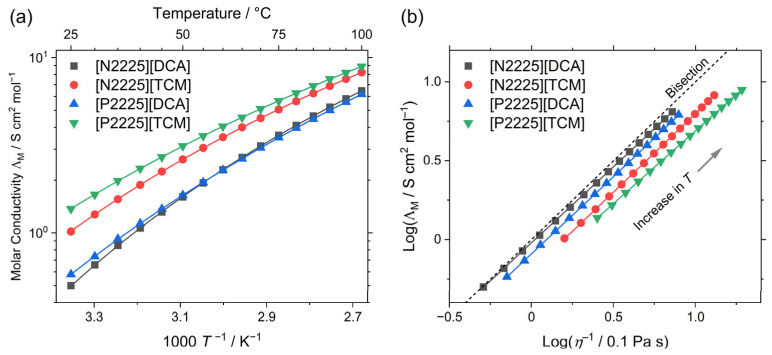
(**a**) Molar conductivity values of the investigated ionic liquids. Drawn lines are the fittings according to the VFT equation (Equation (1)). (**b**) Walden plot of the ionic liquids, including the bisection, which is often termed the ‘ideal KCl line.’ Drawn lines are the linear fits.

The conductivity of ionic liquids is a central quality for their use as electrolytes in electrochemical applications. The obtained values for the molar conductivity $\Lambda_{M}$, the VFT fitting parameters for the temperature-dependent molar conductivity, and Angell’s strength factor are reported in Table 3 and plotted in Figure 4a. Experimental values for the specific conductivity, and calculated values for the molar conductivity as well as activation energy at 25 °C according to the Arrhenius equation, are given in the Supplementary Materials (Table S4, Table S5 and Table S6 respectively)

The molar conductivity at 25 °C shows the exact inverse order of the viscosity, so the values of the [P2225]^+^ samples are higher than those of the [N2225]^+^ samples for the same anion, and the [TCM]^−^ anions give higher conductivities than the [DCA]^−^ samples. The order of the molar conductivity is maintained upon increasing the temperature, with the samples [N2225][DCA] and [P2225][DCA] exchanging their positions around 60 °C. This is the result of the different temperature dependence of the two [DCA] samples. The values for Angell’s strength parameter for the molar conductivity $\delta_{\Lambda_{M}}$ are very similar to the ones obtained for the viscosity $\delta_{\eta }$, showing higher fragilities for the [N2225]^+^ cation and for the [TCM]^−^ anion.

This behavior can be rationalized by the fact that two transport properties are interrelated by the Walden equation $\Lambda_{M}\propto {\left(\eta^{-1}\right)}^{-t}$, with t being a fractional exponent that obtains values close to unit [46,47]. The linear relationship of the two transport properties is illustrated by the Walden plot (Figure 4b). The values for the exponent t range from 0.92 to 0.99, and are thus in the range commonly found for ionic liquids [46,47]. As all samples are very close to the bisection in the Walden plot (often termed the ‘ideal KCl line’), they are classified as ‘good ionic liquids.’ Although this classification is somewhat arbitrary [48], it still allows for comparison with other ionic liquids and electrolytes. For instance, 1-alkyl-1-methylpyrrolidinium cations with the [DCA]^−^ anions have been reported to show values closer to the Walden bisection than other ionic liquids [49].

### 2.4. Self-Diffusion Coefficients

**Figure 5:**
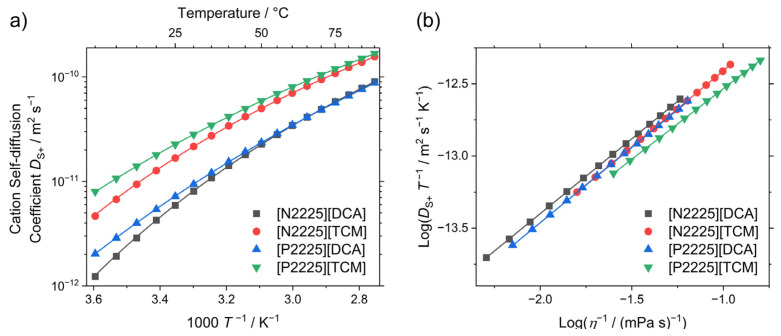
(**a**) Experimental cation self-diffusion coefficients of the investigated ionic liquids. Drawn lines are the fittings according to the VFT equation (Equation (1)). (**b**) Stokes–Einstein plot of the ionic liquids. Drawn lines are the linear fittings.

Compared to the macroscopic properties (viscosity and conductivity), the self-diffusion coefficients give insight into the dynamics on the molecular scale. The cation self-diffusion coefficients $D_{S+}$, as well as the VFT fitting parameters and Angell’s strength parameters for the T-dependence of the cation-self diffusion coefficients, are given in Table 4. The values are plotted in Figure 5a. The experimental values and Arrhenius activation energies at 25 °C are given in the Supplementary Materials (Table S7, Table S8 and Table S9 respectively).

At ambient temperature, the values of the cation self-diffusion coefficients have the same order as the molar conductivity and are inverse to the viscosity. Consequently, the [P2225]^+^ samples have larger self-diffusion coefficients than the [N2225]^+^ samples, and the cations combined with [TCM]^−^ diffuse faster than the ones paired with [DCA]. This order of [P2225][TCM] > [N2225][TCM] > [P2225][DCA] > [N2225][DCA] is maintained in the investigated temperature range, with the two [DCA]^−^ samples exchanging their positions at approximately 65 °C. This crossing of the values for the self-diffusion coefficients at a particular temperature is similar to the behavior of the molar conductivity. By investigating the self-diffusion coefficients, the macroscopic transport behavior can be explained by the larger increase in self-diffusion for the cation of [P2225][DCA] with temperature.

The fragility of the cation self-diffusion coefficients with temperature is similar to the other transport properties. Thus, the samples with the ammonium cation are more fragile than the isostructural phosphonium analogues, and the tricyanomethanide samples have lower $\delta_{D_{S+}}$ values than the dicyanamide ionic liquids with the same cation. The absolute values for Angell’s strength parameter are higher for the cation self-diffusion coefficients than for the macroscopic transport properties (viscosity and molar conductivity).

The self-diffusion coefficients are related to the viscosity by the Stokes–Einstein relation $D_{S}\propto {\left(\eta^{-1}\right)}^{-u},$ with u being a fractional exponent close to unity. The linear relationship between the cation self-diffusion coefficients and the viscosity is shown in Figure 5b. The values for the exponent *u* range from 0.98 to 1.06.

### 2.5. DMA Measurements

**Figure 6:**
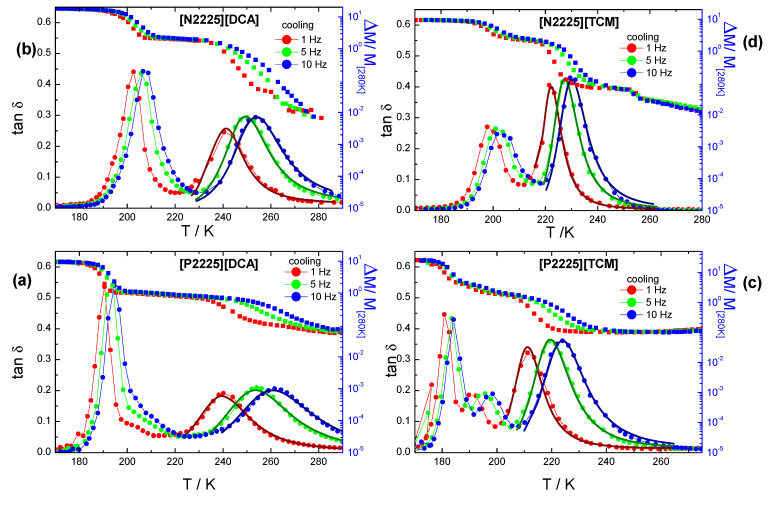
DMA spectra of the samples [P2225][DCA] (**a**), [N2225][DCA] (**b**), [P2225][TCM] (**c**), and [N2225][TCM] (**d**), measured upon cooling at three frequencies (blue, 10 Hz; green, 5 Hz; and red, 1 Hz). The continuous thick line is a fit according to Equations (6)–(8) for the thermally activated peak.

The low-frequency mechanical spectroscopy experiments performed by DMA enable measurement of the mechanical modulus of the ionic liquids and its variation during the main phase transitions occurring by varying the temperature in both the liquid and the solid states. Figure 6 reports the DMA spectra (modulus, M, and tan δ) of the [P2225][DCA], [P2225][TCM], [N2225][DCA], and [N2225][TCM] samples. The storage modulus is plotted as the relative variation with respect to the value measured around room temperature, because it is not possible to separate the contribution of the ILs from that of the pocket, which is considered as a background [10,11].

The spectra measured upon cooling of the four samples are qualitatively similar because they display the same features. In particular, all the samples show the occurrence of a thermally activated relaxation process, which appears at a different temperature for each of them: for a vibration frequency of 1 Hz, it is detectable around 240 K for the two [DCA]-containing ILs, at 210 K for [P2225][TCM], and at 225 K for [N2225][TCM].

Indeed, at these temperatures, the tan δ curve shows a peak; its maximum shifts to a higher temperature with increasing frequency, and, concomitantly, the modulus curve displays a step.

Upon further cooling, all the samples show an intense stiffening of the modulus and an intense peak in tanδ, which displays a limited shift with the frequency. These last features indicate the occurrence of the glass transition, which is detected around 190 K for [P2225][DCA], around 200 K for [N2225][DCA], at 180 K for [P2225][TCM], and around 200 K for [N2225][TCM]. The obtained temperature values are in agreement with those previously obtained by DSC and further confirm that all the liquids undergo a transition to a glass phase.

To obtain information about the dynamic process giving rise to the observed relaxation peak, the data measured at the three frequencies were fitted for each sample using Equation (7), which is appropriate for jumps in an asymmetrical potential well with asymmetry ΔE, and assuming for the relaxation time (τ) a Vogel–Fulcher–Tammann-type (VFT) temperature dependence (Equation (6) or (1)). This model is the same as previously used to fit similar relaxation processes found in the liquid phase of other ILs [9,10,11,12]. The values of the best-fit parameters are reported in Table 5. The values obtained for the *B* and *T_0_* parameters are comparable with those obtained from the VFT fitting of the transport properties and of the diffusion coefficient. In the present case,*B* represents the activation energy measured in K. In all cases, the asymmetry Δ*E* was found to be zero. This last fact is consistent with the observation that in the spectra, the intensity of the relaxation peaks after background subtraction does not increase with the frequency. In particular, the background shift was slightly higher for the curve measured at 1 Hz. Overall, for all the samples, the value obtained for Δ*E* indicates that the relaxation involves two sites with the same energy. An evaluation of the energy barrier that the relaxing unit has to overcome to go from one configuration to the other one is easily obtained by the *B* parameter. 

For all the samples, the obtained energy barriers are rather small, ranging between 452 K (3.6 kJmol^−1^) obtained for the [N2225][TCM] and 1271 K (10.6 kJmol^−1^) obtained for [P2225][DCA]. These values are lower than those usually reported by DMA analyses on other ILs with flexible anions, but closer to that obtained for 1-butyl-3-methyl imidazolium tetracyanoborate [C_4_C_1_im][B(CN)_4_]. Similarly, the relaxation time is in the order of tenths of microseconds and is much larger than those obtained for other ILs. Again, it is close to that obtained for [C_4_C_1_im][B(CN)_4_] [9], as its dynamics are dominated by a mechanism of an intermolecular nature. As previously stated, the obtained *B* parameters are in agreement with those obtained for the VFT fitting of other quantitiessuch as conductivity, viscosity, and cation self-diffusion coefficient. In particular, in all cases, the corresponding energy barrier values obtained for [N2225][DCA] and [P2225][TCM] are very close, while the highest and lowest energies are displayed by [P2225][DCA] and [N2225][TCM], respectively. It must be pointed out that these energy barrier values imply a temperature dependence and are obviously different to those obtained at a certain temperature by a local approximation applying the Arrhenius low, which is not valid to describe the energy behavior in the whole temperature range.

The values obtained for τ_0_ are indicative of a diffusive process, as already reported for some ionic liquids, where the structural relaxation is not dominated by anion flexibility [9,10,11,12]. Moreover, the obtained energy values are quite small, ranging between 3.6 and 10.6 kJmol^−1^, and suggest that the mechanism dominating the observed dynamics is likely of an intermolecular nature [9].

Indeed, contrarily to what is observed in other quaternary ILs with non-flexible anions [12], in the presently studied samples, the presence of rigid anions does not induce the occurrence of at least partial solidification of the samples upon cooling, and this allows the observation of a relaxation process in the supercooled liquid phase. The relaxations observed in these cases, moreover, present parameters very close to those reported by other transport techniques, such as viscosity, conductivity, and self-diffusion coefficients. These observations suggest that in the present case, all these techniques report the same dynamic process. In particular, the similarity between DMA and viscosity VFT analysis parameters indicates that the two techniques provide consistent results regarding the diffusive process dominating the viscous flow, even though they measure a different quantity in different conditions. In fact, as previously stated, the stress applied on the samples during DMA experiments is not a pure shear stress [10], thus allowing the detection of relaxations that are not necessarily observed by applying a pure shear deformation, as in the case of classic shear viscosity measurements. Moreover, it is worth noting that, similarly to what was already observed in the case of [C_4_C_1_im][B(CN)_4_], when the local dynamics are not dominated by the anion flexibility, the DMA data provide indications about the diffusive processes involved in the transport properties. In particular, previous mechanical spectroscopy measurements on ILs with imidazolium cations and anions with different flexibility [9] showed that the fast structural reorganization of flexible anions on a local level affects the dynamics and results in the observation of a relaxation strongly affected by the anion flexibility, which can provide alternative pathways for relaxation on an intermediate timescale. In this framework, the occurrence of translational motion by means of hopping processes (as suggested by the model used for the DMA data) is possibly coupled to the rotational motions and to the transport properties.

### 2.6. Ab Initio Simulations

Throughout the experimental methods used in this work, activation energies tend to be higher for [DCA]^−^ ionic liquids compared to [TCM]^−^ ionic liquids—this was found to be the case for DMA, viscosity, conductivity, and diffusion data. This observation inspired us to perform additional ab initio calculations to gain insight into plausible underlying molecular mechanisms. Specifically, we calculated ion pair complexation energies in the gas phase at two levels of theory (Table 6). For the sake of simplicity, the tetramethyl substituted cations were used as model systems.

The difference in complexation energy between the pairs with [N1111]/[P1111] cations but the same anion is negligible. However, the complexation energies for pairs with the same cation but different anions are much more negative for [DCA]^−^ compared to [TCM]^−^. Specifically, [DCA]^−^ complexes are between 8.2 and 8.8 kcal/mol more stable than [TCM]^−^ complexes. This corresponds to 34 and 37 kJ/mol, or approximately 14 times RT (14 × R × 298 ≈ 35 kJ/mol). The largest part of this stabilizing energy stems from electrostatic interactions (see also Table 6).Thus, it seems plausible that the larger activation energy in [DCM]^−^-based ionic liquids is simply a result of the smaller size of this anion and the resulting higher charge density that dominates the intermolecular interactions and, thus, the structural relaxation.

## 3. Materials and Methods

Details on the synthesis of the investigated ionic liquids and their bromide precursors, including their NMR characterization, are given in the Supplementary Materials. Prior to each physicochemical measurement, the ionic liquids were dried for at least two days in high vacuum. Samples were then handled using Schlenk techniques with argon to avoid the uptake of moisture from the ambient atmosphere. Experimental values for the temperature-dependent density, viscosity, specific conductivity, molar conductivity, and cation self-diffusion coefficients are given in the Supplementary Materials.

### 3.1. DSC Measurements

DSC measurements were performed on a STARe1 DSC (Mettler Toledo, Gießen, Germany) equipped with a nitrogen cooling unit. Approximately 10 mg of the samples were hermetically sealed in aluminum crucibles inside a glove box. The samples were heated from 25° to 125 °C with a heating rate of +5 °C/min to remove the thermal history. Afterwards, the samples were cooled at −1 °C/min down to −120 °C followed by a 5 min isothermal step and subsequent heating to 125 °C with a heating rate of +1 °C/min. The glass transitions are reported by the midpoint method, while the transitions with latent heat are given as the maximum of the thermal event.

### 3.2. Density

Density *ρ* was measured using a pycnometer with 5 mL nominal volume calibrated with pure water and octane. The pycnometer was filled with the ionic liquid and placed in the bath of a Proline 1845 thermostat (LAUDA, Lauda-Königshofen, Germany). After thermal equilibration, the ionic liquid (with *T*-stability better than ±0.01 K) was adjusted to the mark of the pycnometer and cooled, and the weight was determined with a high-precision balance. The density was then calculated from the determined mass and the volume obtained by the calibration. The procedure was repeated in steps of 10 K to construct the *T*-dependent density curves.

### 3.3. Viscosity

Viscosity *η* was measured by stress-controlled rheology using a Physica MCR301 rheometer (Anton Paar, Graz, Austria) with cone-plate geometry (cone diameter of 50 mm) and inert gas flow to avoid uptake of ambient moisture. After thermal equilibration, the viscosity was determined with shear rates that varied from 50 to 150 s^−1^ in linear steps (30 measurement points, each 15 s). As there was no shear rate or time-dependent flow behavior observable (Newtonian fluid behavior), the viscosity values for each temperature were averaged. The process was repeated in 5 K steps, starting from 298.15 K to 378.15 K, to construct the temperature-dependent viscosity curves. The temperature stability during the measurements was better than ±0.01 K. An uncertainty of the viscosity values of ±1.5% was estimated by repeated measurements as well as comparison of the obtained values to commercial (temperature-dependent) viscosity standards and values from the literature for other ionic liquids.

### 3.4. Fitting of the Transport Properties

The temperature-dependent curves of the transport properties Y were fitted using the Vogel–Fulcher–Tammann (VFT) Equation (1).

(1) $$Y=Y_{0}\cdot \exp\left(\frac{B_{Y}}{T-T_{0,Y}}\right)$$

with $Y_{0}$, $B_{Y}$, and $T_{0,Y}$ (Vogel temperature) being empirical fitting parameters. As the viscosity of ionic liquids decreases with the temperature, $B_{\eta }$ takes positive values; meanwhile, conductivity and self-diffusion coefficients increase with the temperature, so negative values are found for $B_{Y}$ in these cases. The ratio of the absolute values of $B_{Y}$ divided by $T_{0,Y}$ is called Angell’s strength factor $\delta_{Y}$ (often termed D in the literature), and it is a measure of the liquid fragility according to the Angell classification [44,47,50].

The activation energy of a particular transport property $E_{a,Y}$ was determined using the Arrhenius-type equation (Equation (2)). Therefore, the slope of the Arrhenius plot ($\ln\left(Y\right)$ vs. $T^{-1}$) at the stated temperature was multiplied with the negative of the gas constant *R*.

(2) $$Y=Y_{0}\cdot \exp\left(-\frac{E_{a,Y}}{RT}\right)$$

### 3.5. Specific and Molar Conductivity

The specific conductivity *κ* was measured by impedance spectroscopy using a SP-150 potentiostat (Biologic, Seyssinet-Pariset, France) in combination with a commercial conductivity probe (WTW, Weilheim, Germany) with a nominal cell constant of 0.5 cm^−1^. The conductivity probe consisted of two rectangular platinum electrodes in parallel orientation fused in glass. The electrodes were freshly platinized before the measurements, and the actual cell constant was determined using commercial conductivity standards at different temperatures. The conductivity cell was filled with the ionic liquids under argon, sealed, and immersed into the bath of a Proline 1845 thermostat (LAUDA, Germany). After temperature equilibration, three impedance measurements with voltage amplitudes of 5, 10, and 15 mV were measured in the frequency range of 200 kHz to 1 Hz in 50 logarithmic steps. From the impedance measurements, the electrolyte resistance was determined, and the results of the three measurements at each temperature were averaged. From the determined electrolyte resistance, the specific conductivity was calculated. The process was repeated from 298.15 K to 373.15 K in steps of 5 K to obtain the temperature-dependent curves of the specific conductivity. The temperature stability during the impedance measurements was better than ±0.01 K. An uncertainty for the specific conductivity values of ±2% was estimated from repeated measurements and the comparison of values obtained this way to commercial conductivity standards and values from the literature of other ionic liquids. The molar conductivity $\Lambda_{M}$ was calculated from the specific conductivity and the density given by Equation (3).

(3) $$\Lambda_{M}=\frac{\kappa }{c}=\frac{\kappa \cdot M}{\rho }$$

with the concentration *c* of the electrolyte and *M* the molar mass of the ionic liquid.

### 3.6. Cation Self-Diffusion Coefficients

The cation self-diffusion coefficients $D_{S+}$ were determined using the pulsed-field gradients stimulated spin echo pulse sequence of NMR spectroscopy utilizing bipolar gradient pulses and longitudinal eddy current delay. Therefore, the dried ionic liquids were placed in the inserts of coaxial NMR tubes (which had an inner diameter of approximately 1 mm) under argon, evacuated, and flame sealed. The narrow tube geometry was chosen to allow for fast thermal equilibration and minimized convection. The measurements were conducted on an Avance 500 Neo (Bruker, Rheinstetten, Germany) with a Prodigy TCI cryo probe head and a BCU II temperature control unit. The temperature unit was calibrated with neat methanol and ethylene glycol [51]. The cation self-diffusion coefficients were determined utilizing the ^1^H signals. Parameter optimization includes the determination of the pulse width, the longitudinal relaxation time $T_{1}$, and a set of diffusion time Δ and gradient duration *δ* that yielded sufficient signal attenuation in the measurements. Therefore, a pair of Δ and *δ* was determined that gave 5% residual signal intensity of the spectrum with the highest field gradient strength g compared to the initial measurement when the gradient was increased from 2 to 95% of the maximum gradient strength (65.7 G cm^−1^ for this setup). The shape of the applied field gradients was that of a smoothed rectangle. The gradient strength was checked by measuring the self-diffusion coefficients of molecular solvent and established ionic liquids and comparing these to values from the literature. With the optimized parameters, a series of 16 measurements with 16 scans were conducted with linearly increased gradient strength from 2 to 95% of the maximum gradient strength. The cation self-diffusion coefficients were obtained by regression of the Stejskal–Tanner Equation (4).

(4) $$I=I_{0}\cdot \exp\left(-D_{S+}{\left(\gamma g\delta \right)}^{2}\left(\Delta -\frac{\delta }{3}\right)\right)$$

with I the signal intensity of the measurement with applied field gradient, $I_{0}$ the initial signal intensity, and γ the gyromagnetic ratio of the nucleus under investigation. An uncertainty of the cation self-diffusion coefficients of ±2% was estimated from repeated measurements, measurements with varied parameters, and the comparison of obtained self-diffusion coefficients to the published values of molecular and ionic liquids.

### 3.7. Dynamical Mechanical Analysis

The Dynamical Mechanical Analysis (DMA) was carried out using a PerkinElmer (Waltham, MA, USA) DMA 8000 instrument by means of a method successfully used in previous studies [9,10,11,12]. Flexural vibration measurements were performed in the three-point bending configuration on a material Pocket supplied by PerkinElmer (30.0 mm by 14.0 mm by 0.5 mm) and filled with the liquid samples. The storage modulus, *M*, and the elastic energy dissipation, tan δ, were measured in an inert nitrogen atmosphere at variable frequencies (1, 5, and 10 Hz, in the present case) and a scanning temperature at 4 K min^−1^ in a range between 160 and 350 K. With this setup, the stress applied on the sample is not a pure shear stress, but, due to the spatial isotropy of liquids, the mechanical modulus presently measured is a combination of both the shear and the bulk modulus [10,11,52,53].

The data were analyzed by means of a two-site model, assuming that the mechanism of relaxation requires a transition between two nonequivalent configurations with an asymmetric potential profile.

When species can move between two configurations with a relaxation rate τ^−1^ by means of thermal activation in a standard anelastic solid [54], the elastic energy dissipation presents a maximum when the Debye relaxation condition, ωτ = 1, is satisfied. For a single relaxation time, τ*,* tan δ is given by:

(5) $$\tan\delta =\Delta \left(T\right)\frac{1}{{\left(\omega \tau \right)}^{-\alpha }+{\left(\omega \tau \right)}^{\alpha }}$$

where ω is the angular vibration frequency and the relaxation intensity, (Δ), is proportional to the concentration of the relaxing species, to the elastic modulus, and to the change in the local distortion, and α is the Fuoss–Kirkwood width parameter and is equal to 1 for a single time Debye relaxation; α < 1 produces broadened peaks with respect to Debye ones.

A Vogel–Fulcher–Tammann-type (VFT) temperature dependence is assumed for the relaxation time τ:

(6) τ=τ0eBkT−T0

It must be noticed that this is the same dependence previously reported in Equation (1), provided that the *B* parameter represents the activation energy and *T*_0_ is the temperature parameter. Indeed, the empirical VFT formula has largely been used to describe the temperature dependence of several physical properties of ionic liquids above the glass transition, such as the conductivity and the inverse of the viscosity, as observed in many other glass-forming liquids [14,15].

If the relaxation occurs between two equivalent sites, the relaxation intensity in Equation (6) decreases with increasing *T*, leading to a higher intensity for the peaks measured at lower frequencies. Instead, in the case of hopping between two nonequivalent configurations with energy separation Δ*E*, the relaxation intensity is proportional to the product of the respective populations in the two configurations, and a more general expression for tan δ is then given by [10,55,56]:

(7) $$\tan\delta =\frac{c}{T\cos h^{2}\left(\Delta E/2kT\right)}\frac{1}{{\left(\omega \tau \right)}^{-\alpha }+{\left(\omega \tau \right)}^{\alpha }}$$

### 3.8. Ab Initio Calculations

Geometry optimizations were performed at the B3LYP-GD3BJ/6-311+G(d,p) level of theory using the Gaussian software package Revision E.01 [57]. Tight geometry convergence criteria were used together with tight SCF convergence criteria (10^−10^ RMS change in the density matrix). Stationary points were confirmed to be true local minimaviathe absence of imaginary frequencies. Counterpoise/BSSE-corrected complexation energies were calculated at the full MP2/cc-pVTZ level of theory with SCF convergence criteria tightened to 10^−11^ RMS change in the density matrix. All calculations in Gaussian were performed using a pruned integration grid with 99 radial shells and 590 angular points per shell, without using symmetry constraints.

SAPT2+/aug-cc-pVDZ (frozen core approximation) calculations were performed using the Psi4 software package, version 1.6.1 [57]. SCF convergence to an energy threshold of 10^−8^ was achieved using a density fitting (DF) algorithm. The Psi4 recipe was followed in calculating and reporting the energy contributions.

## Figures and Tables

**Table 1:** Thermal transitions $T_{X}$ (G: glass transition; CC: cold crystallization upon heating; SS: solid–solid transition; M: melting) obtained by differential scanning calorimetry, and density ρ at 25 °C of the investigated ionic liquids.

	$T_{G}[K]$	$T_{CC}[K]$	$T_{SS}[K]$	$T_{M}[K]$	$\rho^{25\;{}^{\circ}C}[g\;{\mathrm{mL}}^{-1}]$
[N2225][DCA]	195	–	–	–	0.9832
[N2225][TCM]	192	–	–	–	0.9589
[P2225][DCA]	183	244	–	264	1.0064
[P2225][TCM]	179	220	273	278	0.9703

**Table 2:** Experimental viscosity η of the ionic liquids at 25 °C, the obtained VFT fitting parameters $\eta_{0}$, $B_{\eta }$, and $T_{0,\eta }$ according to (Equation (2)) for the temperature-dependent viscosity, and Angell’s factor for the viscosity $\delta_{\eta }$.

	$\eta^{25\;{}^{\circ}C}[\mathrm{mPa}\;s]$	$\eta_{0}[10^{-1}\;\mathrm{mPa}\;s]$	$B_{\eta }\;[K]$	$T_{0,\eta }[K]$	$\delta_{\eta }$
[N2225][DCA]	196.7 ± 3.0	3.067 ± 0.053	695.3 ± 4.3	190.5 ± 0.4	3.65 ± 0.02
[N2225][TCM]	63.0 ± 0.9	3.852 ± 0.076	545.2 ± 5.0	191.2 ± 0.6	2.85 ± 0.03
[P2225][DCA]	141.3± 2.1	1.780 ± 0.098	881.7 ± 16.7	166.1 ± 1.4	5.31 ± 0.11
[P2225][TCM]	39.8 ± 0.6	1.615 ± 0.054	710.7 ± 10.2	169.1 ± 1.1	4.20 ± 0.07

**Table 3:** Molar conductivity $\Lambda_{M}$ of the ionic liquids at 25 °C, the obtained VFT fitting parameters $\Lambda_{M,0}$, $B_{\Lambda_{M}}$, and $T_{0,\Lambda_{M}}$ according to (Equation (1)) for the temperature-dependent molar conductivity, and Angell’s factor for the molar conductivity $\delta_{\Lambda_{M}}$.

	$\Lambda_{M}^{25\;{}^{\circ}C}[S\;{\mathrm{cm}}^{2}\;{\mathrm{mol}}^{-1}]$	$\Lambda_{M,0}[S\;{\mathrm{cm}}^{2}\;{\mathrm{mol}}^{-1}]$	$B_{\Lambda_{M}}[K]$	$T_{0,\Lambda_{M}}[K]$	$\delta_{\Lambda_{M}}$
[N2225][DCA]	0.501 ± 0.01	263.6 ± 6.5	–680.5 ± 7.6	189.7 ± 0.9	3.59 ± 0.04
[N2225][TCM]	1.017 ± 0.02	203.4 ± 4.0	–611.1 ± 6.3	182.7 ± 0.8	3.34 ± 0.04
[P2225][DCA]	0.580 ± 0.01	391.3 ± 10.6	–856.8 ± 9.6	166.6 ± 1.0	5.14 ± 0.07
[P2225][TCM]	1.372 ± 0.03	234.9 ± 5.0	–676.2 ± 7.5	166.6 ± 1.0	4.06 ± 0.05

**Table 4:** Experimental cation self-diffusion coefficients $D_{S+}$ of the ionic liquids at 25 °C, the obtained VFT fitting parameters $D_{S+,0}$, $B_{D_{S+}}$, and $T_{0,D_{S+}}$ according to (Equation (1)) for the temperature dependent cation self-diffusion coefficients, Angell’s factor for the viscosity $\delta_{D_{S+}}$.

	$D_{S+}^{25{}^{\circ}C}[10{-}^{12}\;m^{2}\;s{-}^{1}]$	$D_{S+,0}[10{-}^{8}\;m^{2}\;s{-}^{1}]$	$B_{D_{S+}}[K]$	$T_{0,D_{S+}}[K]$	$\delta_{D_{S+}}$
[N2225][DCA]	5.89 ± 0.12	1.295 ± 0.080	–914.7 ± 19.8	179.1 ± 1.7	5.11 ± 0.12
[N2225][TCM]	16.75 ± 0.34	1.029 ± 0.048	–784.2 ± 14.9	175.9 ± 1.5	4.46 ± 0.09
[P2225][DCA]	7.18 ± 0.14	2.589 ± 0.262	–1215.4 ± 37.8	149.6 ± 2.9	8.12 ± 0.30
[P2225][TCM]	22.63 ± 0.45	1.255 ± 0.058	–887.4 ± 16.2	157.6 ± 1.6	5.63 ± 0.12

**Table 5:** Best-fit parameters obtained for the relaxation processes in the four ILs. In all cases, the value obtained for Δ*E* was zero.

	τ_0_ [s]	T_0_ [K]	α	B [K]
[N2225][DCA]	(4.5 ± 2.7) 10^−7^	180 ± 4	0.8	775 ± 85
[N2225][TCM]	(4.4 ± 0.1) 10^−7^	186 ± 1	0.86	452 ± 220
[P2225][DCA]	(4.0 ± 2) 10^−7^	141 ± 1	0.85	1271 ± 405
[P2225][TCM]	(8.8 ± 5.3) 10^−7^	156 ± 3	0.98	661 ± 69

**Table 6:** Complexation energies in kcal/mol, calculated at the full MP2/cc-pVTZ (counterpoise-corrected) and the SAPT2+/aug-cc-pVDZ level of theory. Both calculation types were performed on geometries optimized at the B3LYP-GD3BJ/6-311+G(d,p) level of theory. The results of the SAPT2+ decomposition are shown in the four rightmost columns.

	MP2	SAPT2+	Electrostatics	Exchange	Dispersion	Induction
[N1111][DCA]	−87.4	−86.1	−90.2	25.2	−11.2	−9.9
[N1111][TCM]	−78.8	−77.9	−79.7	21.4	−10.4	−9.2
[P1111][DCA]	−88.7	−87.5	−91.6	26.5	−12.4	−10.1
[P1111][TCM]	−79.9	−79.0	−80.8	22.7	−11.5	−9.3

## Data Availability

Data are contained within the article or Supplementary Materials.

## Supplementary Materials

The following supporting information can be downloaded at: https://www.mdpi.com/article/10.3390/ijms241311046/s1.

## References

[1] Welton T. (2018). Ionic liquids: A brief history. Biophys. Rev..

[2] Plechkova V., Seddon K.R. (2008). Applications of Ionic Liquids in the Chemical Industry. Chem. Soc. Rev..

[3] Matic A., Scrosati B. (2013). Ionic Liquids for Energy Applications. MRS Bull..

[4] Armand M., Endres F., MacFarlane D.R., Ohno H., Scrosati B. (2011). Ionic-liquid materials for the electrochemical challenges of the future. Materials for Sustainable Energy.

[5] Rauber D., Hofmann A., Philippi F., Kay C.W.M., Zinkevich T., Hanemann T., Hempelmann R. (2021). Structure-Property Relation of Trimethyl Ammonium Ionic Liquids for Battery Applications. Appl. Sci..

[6] Brutti S., Simonetti E., De Francesco M., Sarra A., Paolone A., Palumbo O., Fantini S., Lin R., Falgayrat A., Künzele M. (2020). Ionic liquid electrolytes for high-voltage lithium-ion batteries with a lithium-rich layered oxide positive electrode Li_1.2_Ni_0.2_Mn_0_._6_O_2_. J. Power Sources.

[7] Philippi F., Welton T. (2021). Targeted modifications in ionic liquids—From understanding to design. Phys. Chem. Chem. Phys..

[8] Philippi F., Pugh D., Rauber D., Welton T., Hunt P. (2020). Conformational design concepts for anions in ionic liquids. Chem. Sci..

[9] Philippi F., Rauber D., Palumbo O., Goloviznina K., McDaniel J., Pugh D., Suarez S., Fraenza C.C., Padua A., Kay C.W.M. (2022). Flexibility is the Key to Tuning the Transport Properties of Fluorinated Imide-Based Ionic Liquids. Chem. Sci..

[10] Palumbo O., Trequattrini F., Vitucci F.M., Paolone A. (2015). Relaxation dynamics and phase transitions in ionic liquids: Viscoelastic properties from the liquid to the solid state. J. Phys. Chem. B.

[11] Palumbo O., Trequattrini F., Appetecchi G., Conte L., Paolone A. (2017). Relaxation dynamics in pyrrolidinium based ionic liquids: The role of the anion conformers. J. Mol. Liq..

[12] Palumbo O., Paolone A., Rauber D., Kay C.W.M., Philippi F., Welton T. (2022). Mechanical spectroscopy study of ionic liquids with quaternary cations: Effects of different conformational flexibility. J. Alloys Compd..

[13] Castiglione F., Moreno M., Raos G., Famulari A., Mele A., Appetecchi G.B., Passerini S. (2009). Structural Organization and Transport Properties of Novel Pyrrolidinium-Based Ionic Liquids with Perfluoroalkyl Sulfonylimide Anions. J. Phys. Chem. B.

[14] Rivera A., Rossler E.A. (2006). Evidence of Secondary Relaxations in the Dielectric Spectra Of Ionic Liquids. Phys. Rev. B.

[15] Shamim N., McKenna G.B. (2010). Glass Dynamics and Anomalous Aging in a Family of Ionic Liquids above the Glass TransitionTemperature. J. Phys. Chem. B.

[16] Nakamura K., Shikata T. (2010). Systematic Dielectric and NMR Study -of the Ionic Liquid 1-Alkyl-3-Methyl Imidazolium. Chem. Phys. Chem..

[17] Sangoro J.J., Crosby T., Kremer F. (2016). Dielectric Properties of Ionic Liquids.

[18] Cosby T., Stachurski C.D., Mantz R.A., Trulove P.C., Durkin D.P. (2023). Elucidating the interplay of local and mesoscale ion dynamics and transport properties in aprotic ionic liquids. Phys. Chem. Chem. Phys..

[19] Amith W.D., Araque J.C., Margulis C.J. (2021). Relationship between the Relaxation of Ionic Liquid Structural Motifs and That of the Shear Viscosity. J. Phys. Chem. B.

[20] Sha M., Ma X., Li N., Luo F., Zhu G., Fayer M.D. (2019). Dynamical properties of a room temperature ionic liquid: Using molecular dynamics simulations to implement a dynamic ion cage model. J. Chem. Phys..

[21] Cheng S., Wojnarowska Z., Musiał M., Paluch M. (2021). Correlation between configurational entropy, excess entropy, and ion dynamics in imidazolium-based ionic liquids: Test of the Adam–Gibbs model. J. Chem. Phys..

[22] Schrödle S., Annat G., MacFarlane D.R., Forsyth M., Buchner R., Hefter G. (2007). High Frequency Dielectric Response of the Ionic Liquid N-Methyl-N-ethylpyrrolidinium Dicyanamide. Aust. J. Chem..

[23] Schrodle S., Annat G., MacFarlane D.R., Forsyth M., Buchner R., Hefter G. (2006). Broadband Dielectric Response Of The Ionic Liquid Nmethyl-N-ethylpyrrolidinium Dicyanamide. Chem. Commun..

[24] Krause C., Sangoro J.R., Iacob C., Kremer F. (2010). Charge Transport and Dipolar Relaxations in Imidazolium-Based Ionic, Liquids. J. Phys. Chem. B.

[25] Cosby T., Vicars Z., Mapesa E.U., Tsunashima K., Sangoro J. (2017). Charge transport and dipolar relaxations in phosphonium-based ionic liquids. J. Chem. Phys..

[26] Amith W.D., Araque J.C., Margulis C.J. (2020). A Pictorial View of Viscosity in Ionic Liquids and the Link to Nanostructural Heterogeneity. J. Phys. Chem. Lett..

[27] Yamaguchi T. (2018). Coupling between the mesoscopic dynamics and shear stress of a room-temperature ionic liquid. Phys. Chem. Chem. Phys..

[28] Fraser K.J., Izgorodina E.I., Forsyth M., Scott J.L., MacFarlane D.R. (2007). Liquids intermediate between “molecular” and “ionic” liquids: Liquid Ion Pairs. Chem. Commun..

[29] Philippi F., Rauber D., Zapp J., Präsang C., Scheschkewitz D., Hempelmann R. (2019). Multiple ether-functionalized phosphonium ionic liquids as highly fluid electrolytes. Chem. Phys. Chem..

[30] Bradaric C.J., Downard A., Kennedy C., Robertsona A.J., Zhou Y. (2003). Industrial preparation of phosphonium ionic liquids. Green Chem..

[31] Fraser K.J., MacFarlane D.R. (2009). Phosphonium-Based Ionic Liquids: An Overview. Aust. J. Chem..

[32] Hofmann A., Rauber D., Wang T.-M., Hempelmann R., Kay C.W.M., Hanemann T. (2022). Novel Phosphonium-Based Ionic Liquid Electrolytes for Battery Applications. Molecules.

[33] Philippi F., Rauber D., Kuttich B., Kraus T., Kay C.W.M., Hempelmann R., Hunt P.A., Welton T. (2020). Ether functionalization, ion conformation and the optimisation of macroscopic properties in ionic liquids. Phys. Chem. Chem. Phys..

[34] Lewandowski A., Swiderska-Mocek A. (2009). Ionic liquids as electrolytes for Li-ion batteries—An overview of electrochemical studies. J. Power Sources.

[35] Scarbath-Evers L.K., Hunt P.A., Kirchner B., MacFarlane D.R., Zahn S. (2015). Molecular features contributing to the lower viscosity of phosphonium ionic liquids compared to their ammonium analogues. Phys. Chem. Chem. Phys..

[36] Lima T.A., Paschoal V.H., Faria L.F.O., Ribeiro M.C.C., Ferreira F.F., Costa F.N., Giles C. (2016). Comparing two tetraalkylammonium ionic liquids. II. Phase transitions. J. Chem. Phys..

[37] Seki S., Hayamizu K., Tsuzuki S., Fujii K., Umebayashi Y., Mitsugi T., Kobayashi T., Ohno Y., Kobayashi Y., Mita Y. (2009). Relationships between center atom species (N, P) and ionic conductivity, viscosity, density, self-diffusion coefficient of quaternary cation room-temperature ionic liquids. Phys. Chem. Chem. Phys..

[38] Tsunashima K., Sugiya M. (2007). Physical and electrochemical properties of low-viscosity phosphonium ionic liquids as potential electrolytes. Electrochem. Commun..

[39] Carvalho P.J., Ventura S.P.M., Batista M.L.S., Schröder B., Gonçalves F., Esperança J., Mutelet F., Coutinho J.A.P. (2014). Understanding the impact of the central atom on the ionic liquid behavior: Phosphonium vs ammonium cations. J. Chem. Phys..

[40] Matsumoto M., Takeuchi K., Inoue Y., Tsunashima K., Yamada H. (2022). Molecular Insight into the Ionic Conduction of Quaternary Ammonium and Phosphonium Cation-Based Ionic Liquids Using Dielectric and Spectroscopy Analyses. J. Phys. Chem. B.

[41] Karimi K., Zarrabeitia M., Mariani A., Gatti D., Varzi A., Passerini S. (2021). Nonfluorinated Ionic Liquid Electrolytes for Lithium Metal Batteries: Ionic Conduction, Electrochemistry, and Interphase Formation. Adv. Energy Mater..

[42] Larriba M., Navarro P., García J., Rodríguez F. (2013). Liquid−Liquid Extraction of Toluene from Heptane Using [emim][DCA], [bmim][DCA], and [emim][TCM] Ionic Liquids. Ind. Eng. Chem. Res..

[43] Angell C.A. (1995). Formation of Glasses from Liquids and Biopolymers. Science.

[44] Harris K.R., Kanakubo M., Kodama D., Makino T., Mizuguchi Y., Watanabe M., Watanab T. (2018). Temperature and Density Dependence of the Transport Properties of the Ionic Liquid Triethylpentylphosphonium Bis(trifluoromethanesulfonyl)amide, [P222,5][Tf2N]. J. Chem. Eng. Data.

[45] Schreiner C., Zugmann S., Hartl R., Gores H.J. (2010). Fractional Walden Rule for Ionic Liquids: Examples from Recent Measurements and a Critique of the So-Called Ideal KCl Line for the Walden Plot. J. Chem. Eng. Data.

[46] Schreiner C., Zugmann S., Hartl R., Gores H.J. (2010). Temperature Dependence of Viscosity and Specific Conductivity of Fluoroborate-Based Ionic Liquids in Light of the Fractional Walden Rule and Angell’s Fragility Concept. J. Chem. Eng. Data.

[47] Harris K.R. (2019). On the Use of the Angell–Walden Equation To Determine the “Ionicity” of Molten Salts and Ionic Liquids. J. Phys. Chem. B.

[48] MacFarlane D.R., Forsyth M., Izgorodina E.I., Abbott A.P., Annat G., Fraser K. (2009). On the concept of ionicity in ionic liquids. Phys. Chem. Chem. Phys..

[49] Sippel P., Lunkenheimer P., Krohns S., Thoms E., Loid A. (2015). Importance of liquid fragility for energy applications of ionic liquids. Sci. Rep..

[50] Van Geet A.L. (1970). Calibration of Methanol Nuclear Magnetic Resonance Thermometer at Low Temperature. Anal. Chem..

[51] Makino W., Kishikawa R., Mizoshiri M., Takeda S., Yao M. (2008). Viscoelastic properties of room temperature ionic liquids. J. Chem. Phys..

[52] Yamaguchi T., Miyake S., Koda S. (2010). Shear relaxation of imidazolium-based room- temperature ionic liquids. J. Phys. Chem. B.

[53] Nowick A.S., Berry B.S., Katz J.L. (1972). Anelastic Relaxation in Crystalline Solids.

[54] Cantelli R., Palumbo O., Paolone A., Jensen C.M., Kuba M.T., Ayabe R. (2007). Dynamics of defects in alanates. J. Alloys Compd..

[55] Palumbo O., Cantelli R., Paolone A., Jensen C.M., Srinivasan S.S. (2005). Motion of point defects and monitoring of chemical reactions in sodium aluminiumhydride. J. Alloys Compd..

[56] Frisch M.J., Trucks G.W., Schlegel H.B., Scuseria G.E., Robb M.A., Cheeseman J.R., Scalmani G., Barone V., Mennucci B., Petersson G.A. (2013). Gaussian, 09, Revision E.01.

[57] Smith D.G.A., Burns L.A., Simmonett A.C., Parrish R.M., Schieber M.C., Galvelis R., Kraus P., Kruse H., Di Remigio R., Alenaizan A.A. (2020). PSI4 1.4: Open-source software for high-throughput quantum chemistry. J. Chem. Phys..

