# Supplementary material for: Dynamics in Quaternary Ionic Liquids with Non-Flexible Anions: Insights from Mechanical Spectroscopy
Source: Int J Mol Sci. 2023 Jul 3;24(13):11046. doi: 10.3390/ijms241311046 (PMC10342076; doi:10.3390/ijms241311046)
Supplement: Supplementary file 1 [file ijms-24-11046-s001.zip › ijms-2470374-supplementary.pdf]

# S1. Synthesis

Triethylamine (99.5%) obtained from Carl Roth (Germany) and 1-bromopentane (99%) from Alfa Aesar (Germany) were distilled prior to use. Acetone (99.9%) from Fisher Scientific (Germany) was dried over 4 Å molecular sieves. Triethylphosphine (99%) from Strem (Germany), acetonitrile (HPLC grade) from Th. Geyer (Germany) as well as sodium dicyanamide (>97%) and sodium tricyanomethanide (98%) from IoLiTec (Germany) were used as received. Quaternization reactions were conducted under argon atmosphere. NMR spectra were recorden on an Avance II 400 MHz nuclear magnetic resonance (NMR) spectrometer (Bruker, Germany). Chemical shifts ($\delta$) are reported in parts per million (ppm) relative to tetramethylsilane (TMS).

## S1.1. Triethylpentylammonium bromide [N2225][Br]

For the synthesis of the quaternary ammonium salt 15.0 mLtriethylamine (10.9 g / 108 mmol / 1.3 eq.) and 10.3 mL 1-bromopentane (12.5 g / 83.1 mmol / 1.0 eq.) were dissolved in 150 mL acetonitrile under argon atmosphere. The reaction mixture was stirred for three days at ambient temperature and the solvent and excess reagent removed by rotary evaporation. After further drying for two days in high vacuum 20.3 g of the product (80.6 mmol / 97% yield) were obtained as colorless solid.

**$^1$H-NMR** (400 MHz, d6-DMSO): $\delta$/ppm = 3.22 (q, $^3J_{HH}$ = 7.2 Hz, 6H, N-**CH$_2$**-CH$_3$), 3.10 (m, 2H, N-**CH$_2$**-CH$_2$), 1.62 – 1.50 (m, 2H, N-CH$_2$-**CH$_2$**), 1.39 – 1.21 (m, 4H, N-(CH$_2$)$_2$-(**CH$_2$**)$_2$), 1.16 (t, $^3J_{HH}$ = 7.2 Hz, 9H, N-CH$_2$-**CH$_3$**), 0.88 (t, $^3J_{HH}$ = 7.2 Hz, 3H, N-(CH$_2$)$_4$-**CH$_3$**).

**$^{13}$C{$^1$H}-NMR** (101 MHz, d6-DMSO): $\delta$/ppm = 56.05 (s, N-**CH$_2$**-CH$_2$), 52.04 (s, N-**CH$_2$**-CH$_3$), 27.99 (s, N-CH$_2$-**CH$_2$**), 21.73 (s, N-(CH$_2$)$_2$-**CH$_2$**), 20.71 (s, N-(CH$_2$)$_3$-**CH$_2$**), 13.84 (s, N-(CH$_2$)$_4$-**CH$_3$**), 7.25 (s, N-CH$_2$-**CH$_3$**).

## S1.2. Triethylpentylammoniumdicyanamide [N2225][DCA]

For the synthesis of the title ionic liquids 9.00 g triethylpentyl ammonium bromide (35.7 mmol / 1.0 eq.) and 4.13 g sodium dicyanamide (46.4 mmol / 1.3 eq.) were dissolved in 200 mL acetone and stirred for 24 hours at ambient temperature. After this the solution was filtered, the solvent was removed by rotary evaporation, and the residue dissolved in 150 mL dichloromethane. The solution was dried over magnesium sulfate, filtered and the solvent removed by rotary evaporation. After drying with stirring for two days on a Schlenk line 8.42 g of the product (35.3 mmol / 99% yield) were obtained as colorless liquid.

**$^1$H-NMR** (400 MHz, d6-DMSO): $\delta$/ppm = 3.22 (q, $^3J_{HH}$ = 7.2 Hz, 6H, N-**CH$_2$**-CH$_3$), 3.09 (m, 2H, N-**CH$_2$**-CH$_2$), 1.63 – 1.51 (m, 2H, N-CH$_2$-**CH$_2$**), 1.40 – 1.23 (m, 4H, N-(CH$_2$)$_2$-(**CH$_2$**)$_2$), 1.16 (t, $^3J_{HH}$ = 7.2 Hz, 9H, N-CH$_2$-**CH$_3$**), 0.88 (t, $^3J_{HH}$ = 7.1 Hz, 3H, N-(CH$_2$)$_4$-**CH$_3$**).

**$^{13}$C{$^1$H}-NMR** (101 MHz, d6-DMSO): $\delta$/ppm = 119.16 (s, N(CN)$_2$), 56.10 (s, N-**CH$_2$**-CH$_2$), 52.07 (s, N-**CH$_2$**-CH$_3$), 28.02 (s, N-CH$_2$-**CH$_2$**), 21.77 (s, N-(CH$_2$)$_2$-**CH$_2$**), 20.72 (s, N-(CH$_2$)$_3$-**CH$_2$**), 13.86 (s, N-(CH$_2$)$_4$-**CH$_3$**), 7.22 (s, N-CH$_2$-**CH$_3$**).

## S1.3. Triethylpentylammoniumtricyanomethanide[N2225][TCM]

For the synthesis of the title ionic liquids 9.00 g triethylpentyl ammonium bromide (35.7 mmol / 1.0 eq.) and 5.25 g sodium tricyanomethanide (46.4 mmol / 1.3 eq.) were dissolved in 200 mL acetone and stirred for 24 hours at ambient temperature. After this the solution was filtered, the solvent was removed by rotary evaporation, and the residue dissolved in 150 mL dichloromethane. The solution was dried over magnesium sulfate, filtered and the solvent removed by rotary evaporation. After drying with stirring for two days on a Schlenk line 9.18 g of the product (35.0 mmol / 98% yield) were obtained as colorless liquid.

**$^{1}$H-NMR** (400 MHz, d6-DMSO): $\delta$/ppm = 3.21 (q, $^{3}J_{HH}$ = 7.2 Hz, 6H, N-**$CH_2$**-$CH_3$), 3.09 (m, 2H, N-**$CH_2$**-$CH_2$), 1.63 – 1.50 (m, 2H, N-$CH_2$-**$CH_2$**), 1.40 – 1.21 (m, 4H, N-$(CH_2)_2$-(**$CH_2$**)$_2$), 1.16 (t, $^{3}J_{HH}$ = 7.2 Hz, 9H, N-$CH_2$-**$CH_3$**), 0.89 (t, $^{3}J_{HH}$ = 7.1 Hz, 3H, N-$(CH_2)_4$-**$CH_3$**).

**$^{13}$C{$^{1}$H}-NMR** (101 MHz, d6-DMSO): $\delta$/ppm = 120.60 (s, C(**CN**)$_3$), 56.08 (s, N-**$CH_2$**-$CH_2$), 52.05 (s, N-**$CH_2$**-$CH_3$), 28.01 (s, N-$CH_2$-**$CH_2$**), 21.76 (s, N-$(CH_2)_2$-**$CH_2$**), 20.71 (s, N-$(CH_2)_3$-**$CH_2$**), 13.83 (s, N-$(CH_2)_4$-**$CH_3$**), 7.19 (s, N-$CH_2$-**$CH_3$**).

## S.1.4. Triethylpentyl phosphonium bromide [P2225][Br]

For the synthesis of the quaternary phosphonium salt 12.5 mL triethyphosphine (10 g / 84.6 mmol / 1.0 eq.)and 13.6 mL 1-bromopentane (16.6 g / 110 mmol / 1.2 eq.) were dissolved in 150 mL acetonitrile under argon atmosphere. The reaction mixture was stirred for three days at ambient temperature and the solvent and excess reagent removed by rotary evaporation. After further drying for two days in high vacuum 23.3 g of the product (82.9 mmol / 98% yield) were obtained as colorless solid.

**$^{1}$H-NMR** (400 MHz, d6-DMSO): $\delta$/ppm = 2.36 – 2.20 (m, 8H, P-$CH_2$), 1.55 – 1.41 (m, 2H, P-$CH_2$-**$CH_2$**), 1.41 – 1.24 (m, 4H, P-$(CH_2)_2$-(**$CH_2$**)$_2$ ), 1.11 (dt, $^{3}J_{PH}$ = 18.0 Hz, $^{3}J_{HH}$ = 7.7 Hz, 9H, P-$CH_2$-**$CH_3$**), 0.86 (t, $^{3}J_{HH}$ = 7.0 Hz, 3H, P-$(CH_2)_4$-**$CH_3$**).

**$^{13}$C{$^{1}$H}-NMR** (101 MHz, d6-DMSO): $\delta$/ppm = 32.19 (d, $^{3}J_{CP}$ = 15.0 Hz, P-$(CH_2)_2$-**$CH_2$**), 21.30 (s, P-$(CH_2)_3$-**$CH_2$**), 20.17 (d, $^{2}J_{CP}$ = 4.4 Hz, P-$CH_2$-**$CH_2$**), 16.79 (d, $^{1}J_{CP}$ = 47.5 Hz, P-**$CH_2$**-$CH_2$), 13.59 (s, P-$(CH_2)_4$-**$CH_3$**), 10.65 (d, $^{1}J_{CP}$ = 48.7 Hz, P-**$CH_2$**-$CH_3$), 5.25 (d, $^{2}J_{CP}$ = 5.4 Hz, P-$CH_2$-**$CH_3$**).

**$^{31}$P{$^{1}$H}-NMR** (162 MHz, d6-DMSO): $\delta$/ppm = 38.88 (s).

## S1.5. Triethylpentyl phosphonium dicyanamide [P2225][DCA]

For the synthesis of the title ionic liquids 9.00 g triethylpentyl phosphonium bromide (33.4 mmol / 1.0 eq.) and 3.87 g sodium dicyanamide (43.4 mmol / 1.3 eq.) were dissolved in 200 mL acetone and stirred for 24 hours at ambient temperature. After this the solution was filtered, the solvent was removed by rotary evaporation, and the residue dissolved in 150 mL dichloromethane. The solution was dried over magnesium sulfate, filtered and the solvent removed by rotary evaporation. After drying with stirring for two days on a Schlenk line 8.44 g of the product (33.1 mmol / 99% yield) were obtained as colorless liquid.

**$^{1}$H-NMR** (400 MHz, d6-DMSO): $\delta$/ppm = 2.28 – 2.12 (m, 8H, P-$CH_2$), 1.55 – 1.42 (m, 2H, P-$CH_2$-**$CH_2$**), 1.42 – 1.26 (m, 4H, P-$(CH_2)_2$-(**$CH_2$**)$_2$ ), 1.13 (dt, $^3J_{PH}$ = 18.0 Hz, $^3J_{HH}$ = 7.6 Hz, 9H, P-$CH_2$-**$CH_3$**), 0.88 (t, $^3J_{HH}$ = 7.0 Hz, 3H, P-$(CH_2)_4$-**$CH_3$**).

**$^{13}$C{$^1$H}-NMR** (101 MHz, d6-DMSO): $\delta$/ppm = 119.14 (s, $N(CN)_2$), 32.33 (d, $^3J_{CP}$ = 15.0 Hz, P-$(CH_2)_2$-**$CH_2$**), 21.44 (s, P-$(CH_2)_3$-**$CH_2$**), 20.23 (d, $^2J_{CP}$ = 4.4 Hz, P-$CH_2$-**$CH_2$**), 16.46 (d, $^1J_{CP}$ = 47.4 Hz, P-**$CH_2$**-$CH_2$), 13.72 (s, P-$(CH_2)_4$-**$CH_3$**), 10.63 (d, $^1J_{CP}$ = 48.9 Hz, P-**$CH_2$**-$CH_3$), 5.25 (d, $^2J_{CP}$ = 5.3 Hz, P-$CH_2$-**$CH_3$**).

**$^{31}$P{$^1$H}-NMR** (162 MHz, d6-DMSO): $\delta$/ppm = 38.93 (s).

## S1.6. Triethylpentyl phosphonium tricyanomethanid [P2225][TCM]

For the synthesis of the title ionic liquids 9.00 g triethylpentyl phosphonium bromide (33.4 mmol / 1.0 eq.) and 4.90 g sodium dicyanamide (43.4 mmol / 1.3 eq.) were dissolved in 200 mL acetone and stirred for 24 hours at ambient temperature. After this the solution was filtered, the solvent was removed by rotary evaporation, and the residue dissolved in 150 mL dichloromethane. The solution was dried over magnesium sulfate, filtered and the solvent removed by rotary evaporation. After drying with stirring for two days on a Schlenk line 9.14 g of the product (32.7 mmol / 98% yield) were obtained as colorless liquid.

**$^{1}$H-NMR** (400 MHz, d6-DMSO): $\delta$/ppm = 2.26 – 2.1i0 (m, 8H, P-$CH_2$), 1.55 – 1.42 (m, 2H, P-$CH_2$-z**$CH_2$**), 1.42 – 1.28zz (m, 4H, P-$(CH_2)_2$-(**$CH_2$**)$_2$ ), 1.11 (dt, $^{3}J_{PH}$ = 18.0 Hz, $^{3}J_{HH}$ = 7.8 Hz, 9H, P-$CH_2$-**$CH_3$**), 0.88 (t, $^{3}J_{HH}$ = 7.1 Hz, 3H, P-$(CH_2)_4$-**$CH_3$**).

**$^{13}$C{$^{1}$H}-NMR** (101 MHz, d6-DMSO): $\delta$/ppm = 120.58 (s, C(**CN**)$_3$), 32.33 (d, $^{3}J_{CP}$ = 14.8 Hz, P-$(CH_2)_2$-**$CH_2$**), 21.45 (s, P-$(CH_2)_3$-**$CH_2$**), 20.23 (d, $^{2}J_{CP}$ = 4.5 Hz, P-$CH_2$-**$CH_2$**), 16.45 (d, $^{1}J_{CP}$ = 47.5 Hz, P-**$CH_2$**-$CH_2$), 13.71 (s, P-$(CH_2)_4$-**$CH_3$**), 10.62 (d, $^{1}J_{CP}$ = 48.9 Hz, P-**$CH_2$**-$CH_3$), 5.22 (d, $^{2}J_{CP}$ = 5.3 Hz, P-$CH_2$-**$CH_3$**).

**$^{31}$P{$^{1}$H}-NMR** (162 MHz, d6-DMSO): $\delta$/ppm = 38.95 (s).

# S2. Physicochemical Characterization

## S2.1 Density

Experimental values of the ionic liquids' densities are given in Table S1. The linear fitting parameter according to equation (S1) are given Table S2.

$$\rho = n - m \cdot T \qquad \text{(S1)}$$

**Table S1:**Experimental $T$-dependent values of the ionic liquids' density $\rho$.

| $T$/ K | Density $\rho$/ g mL$^{-1}$. | | | |
|---|---|---|---|---|
| | [N2225][DCA] | [N2225][TCM] | [P2225][DCA] | [P2225][TCM] |
| 298.15 | 0.9832 | 0.9589 | 1.0064 | 0.9703 |
| 308.15 | 0.9775 | 0.9530 | 1.0008 | 0.9645 |
| 318.15 | 0.9716 | 0.9472 | 0.9950 | 0.9584 |
| 328.15 | 0.9661 | 0.9415 | 0.9897 | 0.9524 |
| 338.15 | 0.9607 | 0.9353 | 0.9839 | 0.9465 |
| 348.15 | 0.9552 | 0.9301 | 0.9785 | 0.9407 |
| 358.15 | 0.9498 | 0.9245 | 0.9730 | 0.9352 |
| 368.15 | 0.9446 | 0.9190 | 0.9675 | 0.9291 |

**Table S2:** Linear fitting parameters$a$ and $b$ for the ionic liquids' densities according to equation (S1).

| Ionic liquid | $n$ / g mL$^{-1}$ | $m$ / $10^{-4}$g mL$^{-1}$K$^{-1}$ | $R^2$ |
|---|---|---|---|
| [N2225][DCA] | 1.147 ± 0.001 | 5.515 ± 0.037 | 0.99973 |
| [N2225][TCM] | 1.129 ± 0.001 | 5.709 ± 0.041 | 0.99969 |
| [P2225][DCA] | 1.172 ± 0.001 | 5.559 ± 0.023 | 0.99989 |
| [P2225][TCM] | 1.146 ± 0.001 | 5.882 ± 0.028 | 0.99986 |

## S2.2. Viscosity

Experimental values of the ionic liquids' viscosity $\eta$ at different temperatures are given in Table S3. The fitting parameters for the Vogel-Fulcher-Tammann equation are given in the main manuscript.

**Table S3:**Experimental $T$-dependent viscosity values of the investigated ionic liquids.

| $T$ / K | Viscosity $\eta$ / mPa s | | | |
|---|---|---|---|---|
| | [N2225][DCA] | [N2225][TCM] | [P2225][DCA] | [P2225][TCM] |
| 298.15 | 196.7 | 63.03 | 141.3 | 39.81 |
| 303.15 | 147.5 | 50.05 | 110.4 | 32.37 |
| 308.15 | 113.6 | 40.72 | 88.24 | 26.72 |
| 313.15 | 89.24 | 33.65 | 71.50 | 22.38 |
| 318.15 | 71.52 | 28.27 | 58.84 | 18.98 |
| 323.15 | 58.10 | 24.00 | 48.95 | 16.31 |
| 328.15 | 48.02 | 20.65 | 41.14 | 14.13 |
| 333.15 | 40.19 | 17.94 | 34.90 | 12.30 |
| 338.15 | 34.03 | 15.73 | 29.67 | 10.82 |
| 343.15 | 29.15 | 13.92 | 25.65 | 9.60 |
| 348.15 | 25.27 | 12.42 | 22.59 | 8.57 |
| 353.15 | 22.07 | 11.15 | 19.74 | 7.70 |
| 358.15 | 19.44 | 10.08 | 17.53 | 6.94 |
| 363.15 | 17.25 | 9.16 | 15.67 | 6.28 |
| 368.15 | 15.43 | 8.38 | 14.03 | 5.70 |
| 373.15 | 13.87 | 7.70 | 12.68 | 5.22 |
| 378.15 | 12.57 | 7.11 | 11.38 | 4.80 |

## S2.3. Specific Conductivity

Experimental values for the specific conductivity$\kappa$ obtained by impedance spectroscopy are given in Table S4. The fitting parameter according to the VFT equation are summarized in Table S5.

**Table S4:**Experimental values for the specific conductivity of the investigated ionic liquids.

| $T$ / K | Specific conductivity $\kappa$ / mS cm$^{-1}$ | | | |
|---|---|---|---|---|
| | [N2225][DCA] | [N2225][TCM] | [P2225][DCA] | [P2225][TCM] |
| 298.15 | 2.065 | 3.687 | 2.286 | 4.730 |
| 303.15 | 2.705 | 4.608 | 2.885 | 5.687 |
| 308.15 | 3.476 | 5.610 | 3.620 | 6.786 |
| 313.15 | 4.360 | 6.750 | 4.422 | 7.953 |
| 318.15 | 5.365 | 8.014 | 5.330 | 9.227 |
| 323.15 | 6.522 | 9.376 | 6.377 | 10.622 |
| 328.15 | 7.816 | 10.86 | 7.523 | 12.095 |
| 333.15 | 9.254 | 12.46 | 8.809 | 13.66 |
| 338.15 | 10.85 | 14.15 | 10.21 | 15.31 |
| 343.15 | 12.59 | 15.92 | 11.72 | 17.08 |
| 348.15 | 14.45 | 17.78 | 13.40 | 18.92 |
| 353.15 | 16.39 | 19.75 | 15.14 | 20.86 |
| 358.15 | 18.49 | 21.84 | 16.98 | 22.87 |
| 363.15 | 20.71 | 23.99 | 19.00 | 24.97 |
| 368.15 | 23.05 | 26.20 | 21.10 | 27.04 |
| 373.15 | 25.50 | 28.49 | 23.34 | 29.25 |

**Table S5:** VFT fitting parameters$\kappa_0$, $B_\kappa$ and$T_{0,\kappa}$for the specific conductivity $\kappa$ of the investigated ionic liquids including coefficient of determination $R^2$.

| Ionic liquid | $\kappa_0$/ mS cm$^{-1}$ | $B_\kappa$ / K | $T_{0,\kappa}$ / K | $R^2$ |
|---|---|---|---|---|
| [N2225][DCA] | 890.9 ± 23.0 | –641.7 ± 7.9 | 192.55 ± 0.9 | 0.99999 |
| [N2225][TCM] | 591.9 ± 9.5 | –566.0 ± 5.0 | 186.62 ± 0.7 | >0.99999 |
| [P2225][DCA] | 1233.3 ± 32.4 | –805.4 ± 9.1 | 170.16 ± 1.0 | >0.99999 |
| [P2225][TCM] | 635.9 ± 12.9 | –621.0 ± 6.9 | 170.16 ± 1.0 | >0.99999 |

## S2.4. Molar conductivity

Calculated values of the ionic liquids' molar conductivity $\Lambda_M$ at different temperatures are given in Table S6. The fitting parameters for the VFT equation are given in the main manuscript.

**Table S6:** Calculated values of the ionic liquids' molar conductivities at different temperatures.

| $T$ / K | Molar conductivity $\Lambda_M$ / S cm$^2$mol$^{-1}$ | | | |
|---|---|---|---|---|
| | [N2225][DCA] | [N2225][TCM] | [P2225][DCA] | [P2225][TCM] |
| 298.15 | 0.501 | 1.017 | 0.580 | 1.372 |
| 303.15 | 0.658 | 1.275 | 0.734 | 1.654 |
| 308.15 | 0.848 | 1.557 | 0.924 | 1.980 |
| 313.15 | 1.066 | 1.878 | 1.132 | 2.328 |
| 318.15 | 1.316 | 2.237 | 1.368 | 2.709 |
| 323.15 | 1.604 | 2.625 | 1.641 | 3.128 |
| 328.15 | 1.928 | 3.051 | 1.941 | 3.573 |
| 333.15 | 2.289 | 3.510 | 2.279 | 4.047 |
| 338.15 | 2.693 | 3.998 | 2.648 | 4.551 |
| 343.15 | 3.132 | 4.513 | 3.050 | 5.092 |
| 348.15 | 3.606 | 5.055 | 3.497 | 5.658 |
| 353.15 | 4.102 | 5.631 | 3.962 | 6.257 |
| 358.15 | 4.641 | 6.246 | 4.457 | 6.883 |
| 363.15 | 5.214 | 6.882 | 5.001 | 7.538 |
| 368.15 | 5.819 | 7.540 | 5.570 | 8.188 |
| 373.15 | 6.455 | 8.225 | 6.177 | 8.888 |

## S2.5. Cation self-diffusion coefficients

Experimental values of the ionic liquids'' cation self-diffusion coefficients $D_{S+}$ at different temperatures are given in Table S7. The fitting parameters for the VFT equation are given in the main manuscript.

**Table S7:** Experimental values for the cation self-diffusion coefficients $D_{S+}$of the ionic liquids

| $T$ / K | Cation self-diffusion coefficient $D_{S+}$ / $m^2 s^{-1}$ | | | |
|---|---|---|---|---|
| | [N2225][DCA] | [N2225][TCM] | [P2225][DCA] | [P2225][TCM] |
| 278.15 | $1.23 \times 10^{-12}$ | $4.65 \times 10^{-12}$ | $2.03 \times 10^{-12}$ | $7.98 \times 10^{-12}$ |
| 283.15 | $1.92 \times 10^{-12}$ | $6.73 \times 10^{-12}$ | $2.87 \times 10^{-12}$ | $1.07 \times 10^{-11}$ |
| 288.15 | $2.88 \times 10^{-12}$ | $9.37 \times 10^{-12}$ | $3.98 \times 10^{-12}$ | $1.40 \times 10^{-11}$ |
| 293.15 | $4.25 \times 10^{-12}$ | $1.27 \times 10^{-11}$ | $5.40 \times 10^{-12}$ | $1.79 \times 10^{-11}$ |
| 298.15 | $5.89 \times 10^{-12}$ | $1.68 \times 10^{-11}$ | $7.18 \times 10^{-12}$ | $2.26 \times 10^{-11}$ |
| 303.15 | $8.06 \times 10^{-12}$ | $2.16 \times 10^{-11}$ | $9.40 \times 10^{-12}$ | $2.82 \times 10^{-11}$ |
| 308.15 | $1.08 \times 10^{-11}$ | $2.74 \times 10^{-11}$ | $1.21 \times 10^{-11}$ | $3.44 \times 10^{-11}$ |
| 313.15 | $1.41 \times 10^{-11}$ | $3.40 \times 10^{-11}$ | $1.54 \times 10^{-11}$ | $4.17 \times 10^{-11}$ |
| 318.15 | $1.81 \times 10^{-11}$ | $4.17 \times 10^{-11}$ | $1.92 \times 10^{-11}$ | $4.97 \times 10^{-11}$ |
| 323.15 | $2.27 \times 10^{-11}$ | $5.00 \times 10^{-11}$ | $2.36 \times 10^{-11}$ | $5.90 \times 10^{-11}$ |
| 328.15 | $2.81 \times 10^{-11}$ | $5.96 \times 10^{-11}$ | $2.87 \times 10^{-11}$ | $6.91 \times 10^{-11}$ |
| 333.15 | $3.42 \times 10^{-11}$ | $7.01 \times 10^{-11}$ | $3.46 \times 10^{-11}$ | $8.01 \times 10^{-11}$ |
| 338.15 | $4.12 \times 10^{-11}$ | $8.17 \times 10^{-11}$ | $4.11 \times 10^{-11}$ | $9.17 \times 10^{-11}$ |
| 343.15 | $4.89 \times 10^{-11}$ | $9.45 \times 10^{-11}$ | $4.87 \times 10^{-11}$ | $1.05 \times 10^{-10}$ |
| 348.15 | $5.79 \times 10^{-11}$ | $1.08 \times 10^{-10}$ | $5.67 \times 10^{-11}$ | $1.19 \times 10^{-10}$ |
| 353.15 | $6.72 \times 10^{-11}$ | $1.23 \times 10^{-10}$ | $6.58 \times 10^{-11}$ | $1.34 \times 10^{-10}$ |
| 358.15 | $7.82 \times 10^{-11}$ | $1.39 \times 10^{-10}$ | $7.61 \times 10^{-11}$ | $1.50 \times 10^{-10}$ |
| 363.15 | $9.01 \times 10^{-11}$ | $1.56 \times 10^{-10}$ | $8.77 \times 10^{-11}$ | $1.67 \times 10^{-10}$ |

## S2.6. Walden Relation

Experimental values of the viscosity and molar conductivity were analyzed using the Walden relation according to equation (S2). The obtained linear fitting parameters are given in Table S8.

$$\log\left(\frac{\Lambda_M}{S \cdot cm^2 \cdot mol^{-1}}\right) = \log(C) + t \cdot \log\left(\frac{0.1\ Pa \cdot s}{\eta}\right) \qquad (S2)$$

**Table S8:** Linear fitting parameters $\log(C)$ and $t$ according to the Walden relation (equation (S2)) including coefficient of determination $R^2$.

| Ionic liquid | $\log(C)$/ $10^{-1}$ | $t$ / 1 | $R^2$ |
|---|---|---|---|
| [N2225][DCA] | –0.198 ± 0.006 | 0.965 ± 0.001 | 0.99998 |
| [N2225][TCM] | –1.952 ± 0.013 | 0.994 ± 0.002 | 0.99996 |
| [P2225][DCA] | –0.899 ± 0.009 | 0.979 ± 0.002 | 0.99996 |
| [P2225][TCM] | –2.319 ± 0.010 | 0.922 ± 0.001 | 0.99998 |

## S2.7. Stokes–Einstein Relation

Experimental values of the viscosity and the cation self-diffusion coefficients were analyzed using the Stokes–Einstein relation according to equation (S3). The obtained linear fitting parameters are given in Table S9.

$$\log\left(\frac{D_{S+}}{\mathrm{m^2 s^{-1}}} \cdot \frac{T^{-1}}{\mathrm{K^{-1}}}\right) = a + u \cdot \log(\eta^{-1}/(\mathrm{mPa\ s})^{-1}) \quad \text{(S3)}$$

**Table S9:** Linear fitting parameters $a$ and $u$ according to the Stokes–Einstein relation (equation (S3)) including coefficient of determination $R^2$.

| Ionic liquid | $a/\,1$ | $u\,/\,1$ | $R^2$ |
|---|---|---|---|
| [N2225][DCA] | −11.324 ± 0.002 | 1.038 ± 0.001 | 0.99998 |
| [N2225][TCM] | −11.355 ± 0.001 | 1.055 ± 0.001 | 0.99999 |
| [P2225][DCA] | −11.374 ± 0.004 | 1.044 ± 0.002 | 0.99994 |
| [P2225][TCM] | −11.554 ± 0.002 | 0.979 ± 0.002 | 0.99997 |
